# High Solid Fluorescence of a Pyrazoline Derivative through Hydrogen Bonding

**DOI:** 10.3390/molecules22081304

**Published:** 2017-08-04

**Authors:** Liang Zhang, Jie Liu, Junkuo Gao, Feng Zhang, Liang Ding

**Affiliations:** 1 School of Material Science and Engineering, Yancheng Institute of Technology, Yancheng 224051, China; zhangliang@ycit.edu.cn (L.Z.); zhangfeng@ycit.cn (F.Z.); 2 State Key Laboratory of Crystal Materials, Shandong University, Shandong 250100, China; liujieicm@sdu.edu.cn; 3 College of Materials and Textiles, Zhejiang Sci-Tech University, Hangzhou 310018, China; jkgao@zstu.edu.cn

**Keywords:** pyrazoline, fluorescence, hydrogen bonding, fluorine, intramolecular rotation

## Abstract

Pyrazoline and its derivatives often exhibit strong emissions in dilute solutions, but their emission intensity is often dramatically reduced in the solid state due to strong intermolecular interactions between neighboring molecules. In this report, we successfully synthesized a new pyrazoline 4-(3-(4-(decyloxy)phenyl)-1-(2,3,5,6-tetrafluoro-4-(trifluoromethyl)phenyl)-4,5-dihydro-1*H*-pyrazol-5-yl)-*N*,*N*-diethylaniline (PPDPD), into which seven fluorine (F) atoms were incorporated. In the solid state, PPDPD emits a strong blue light at λ_max_ 430 nm with a fluorescence quantum yield of up to 41.3%. Single-crystal analysis showed the presence of intra/intermolecular C-H···F bonds that may impede molecular motion and block the non-radiative decay channel. Compound PPDPD therefore shows high emission efficiency in the solid state.

## 1. Introduction

Amongst the numerous heterocyclic organic luminescent materials, pyrazoline and its derivatives have received increasing attention due to their excellent electro-optical properties [1,2,3,4,5,6,7,8,9,10,11,12,13,14] and wide range of applications. For example, they are used in fluorescent probes, organic light-emitting diodes, and many other materials [15,16,17,18,19,20,21,22,23,24]. Generally, pyrazoline and its derivatives display strong emission in dilute solutions, but their emission efficiencies can dramatically decrease in the solid state (e.g., in high concentration in solution, in the aggregated state, as a powder, film, or single crystal) due to strong intramolecular and/or intermolecular interactions between neighboring molecules. Thus, the development of a pyrazoline backbone that possesses high emission efficiency in the solid state is greatly desired. In order to overcome these problems, Gu and co-workers introduced a single aggregation-induced emission (AIE) unit based on a pyrazoline backbone into the polymer chain via atom-transfer radical polymerization [25]. As a result, a series of AIE-terminated vinyl polymers bearing different polarities were successfully synthesized. The fluorescent quantum yield of an AIE-terminated polystyrene can reach 48%. To the best of our knowledge, most of the current research in this area is focused on AIE-terminated or AIE polymers; small molecules based on a pyrazoline backbone have not been fully investigated [26,27]. Compared to polymers based on a pyrazoline backbone, small molecules provide several advantages, such as monodispersity, reliable synthetic reproducibility, and adjustable structure packing features.

**Scheme 1:**
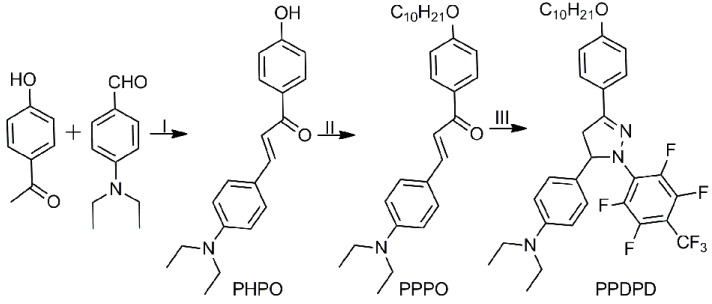
Synthetic route to compound 4-(3-(4-(decyloxy)phenyl)-1-(2,3,5,6-tetrafluoro-4-(trifluoromethyl)phenyl)-4,5-dihydro-1*H*-pyrazol-5-yl)-*N*,*N*-diethylaniline (PPDPD). (I) H_2_SO_4_/CH_3_COOH, 75%; (II) 1-bromodecane/potassium carbonate/acetone, 72%; (III) (2,3,5,6-tetrafluoro-4-(trifluoromethyl)phenyl)hydrazine/HCl/ethanol, 76%.

Herein, we successfully synthesized a new pyrazoline 4-(3-(4-(decyloxy)phenyl)-1-(2,3,5,6-tetrafluoro-4-(trifluoromethyl)phenyl)-4,5-dihydro-1*H*-pyrazol-5-yl)-*N*,*N*-diethylaniline (PPDPD, Scheme 1), into which seven fluorines (F) were incorporated. We believe that the intermolecular interactions between neighboring molecules of the PPDPD compound may differ from those present in commonly-used pyrazoline and its derivatives due to the influence of the C-H···F [28,29] hydrogen bond. Furthermore, the presence of this interaction may hinder the intramolecular rotation process of PPDPD in the solid state; this could block the non-radiative decay channel and hence improve its emission properties.

## 2. Results and Discussion

### 2.1. Synthesis of Compound PPDPD

The synthetic procedure for the preparation of compound PPDPD is shown in Scheme 1. The α,β-unsaturated ketone 3-(4-(diethylamino)phenyl)-1-(4-hydroxyphenyl)prop-2-en-1-one (PHPO) was prepared in 75% yield via the reaction of 4-diethylaminobenzaldehyde and 4′-hydroxyacetophenone under acidic conditions. To increase the solubility and reduce the π-π stacking interactions, decane was introduced into the molecular backbone to give 1-(4-(decyloxy)phenyl)-3-(4-(diethylamino)phenyl)prop-2-en-1-one (PPPO). Subsequently, the target compound PPDPD was prepared in 76% yield via the reaction of PPPO with (2,3,5,6-tetrafluoro-4-(trifluoromethyl)phenyl)hydrazine in the presence of acid and under an N_2_ atmosphere. The target compound PPDPD was characterized using ^1^H, ^13^C, and ^19^F NMR spectroscopy, mass spectrometry, and single crystal analysis.

### 2.2. Optical Properties of Compound PPDPD

**Figure 1:**
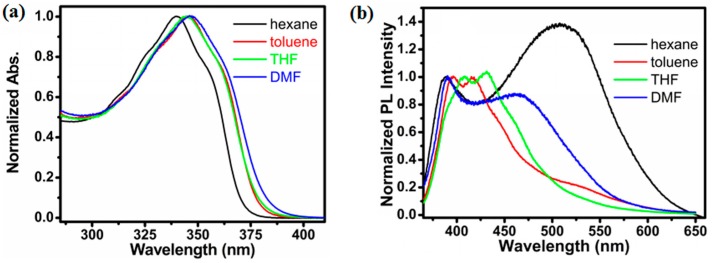
Normalized (**a**) absorption and (**b**) fluorescence emission spectra of compound PPDPD in different dilute solutions. DMF: dimethylformamide; THF: tetrahydrofuran.

The normalized optical absorption and emission spectra of compound PPDPD in different polar solvents are shown in Figure 1. In the non-polar solvent hexane, PPDPD exhibits one main absorption band at λ_max_ 340 nm (Figure 1a). Compared to the optical absorption behavior in hexane, the main absorption bands at λ_max_ in solvents of moderate polarity (e.g., toluene and tetrahydrofuran (THF)) are red-shifted by 5 nm. In the polar solvent dimethylformamide (DMF), λ_max_ is further red-shifted to 346 nm. The photoluminescent (PL) behaviors of PPDPD recorded in different polar solvents are shown in Figure 1b. The emission spectrum of PPDPD in the non-polar solvent hexane showed two main emission bands at λ_max_ 389 nm and 505 nm. This differs from the commonly-used 1,3,5-triphenyl-2-pyrazoline, which emits a strong blue light with one main emission band at a λ_max_ of approximately 460 nm [30,31,32,33]. Moreover, the fluorescence quantum yield of PPDPD in hexane is below 1% using quinine bisulfate (ϕ = 54.6% in 0.1 N H_2_SO_4_) as a standard. Generally, these phenomena may be ascribed to the fact that compound PPDPD forms an excimer in hexane [3,34]. To confirm this hypothesis, the emission behaviors of different concentrations of PPDPD hexane solutions were analyzed (Figure S5). Compound PPDPD exhibits two main emission bands at λ_max_ 389 nm and 450 nm at a concentration of 10^−3^ mol L^−1^. Although no significant change in the shorter wavelength (389 nm) was observed following an increase in concentration, the longer wavelength was red-shifted to 505 nm at a 10^−4^ mol L^−1^ concentration. Moreover, the PL intensity at 505 nm decreased following a reduction in concentration. The emission behaviors (depending on the concentration) of PPDPD in hexane at the longer wavelength may be due to the poor solubility of PPDPD. Aggregation may differ at high concentrations such as 10^−3^ and 10^−4^ mol L^−1^. When the solvent is changed from the non-polar hexane to the moderately polar toluene/THF, the shorter wavelength is slightly red-shifted. Changing from the moderately polar toluene/THF to the polar DMF results in the longer wavelength being red-shifted. This may be due to a charge-transfer (CT) interaction between the electron donor groups and the electron acceptor groups because CT processes are easily affected by solvents.

**Figure 2:**
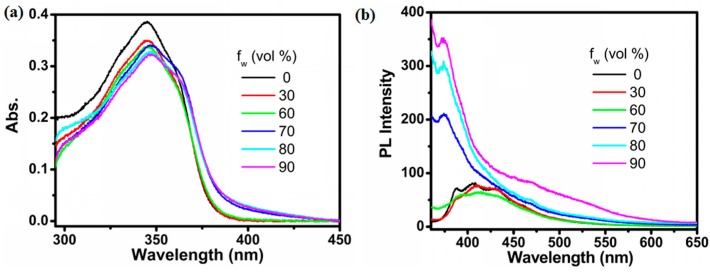
(**a**) Absorption and (**b**) fluorescence emission spectra of compound PPDPD in the THF/water mixtures (10^−5^ mol L^−1^).

The fluorescence quantum yield of compound PPDPD in common solvents (hexane, toluene, THF, and DMF) is also below 1% with quinine bisulfate (ϕ = 54.6% in 0.1 N H_2_SO_4_) as a standard. We further investigated the effect of aggregation on the fluorescence of PPDPD. The optical absorption and emission spectra of PPDPD in a THF/water mixture are displayed in Figure 2. The λ_max_ was red-shifted following an increase in the water fraction (f_w_). When the f_w_ value reached 70%, leveling-off tails in the long absorption spectra can be observed, suggesting that nanoparticles are formed at a high f_w_ value [35,36,37]. The emission spectra of PPDPD in a THF/water mixture is shown in Figure 2b. At a low f_w_ value and increasing from 0 to 60%, no obvious change in PL intensity and wavelength are observed. However, due to the presence of aggregates, an increase in PL intensity and a blue-shift in the PL wavelength is observed when the f_w_ value is increased.

**Figure 3:**
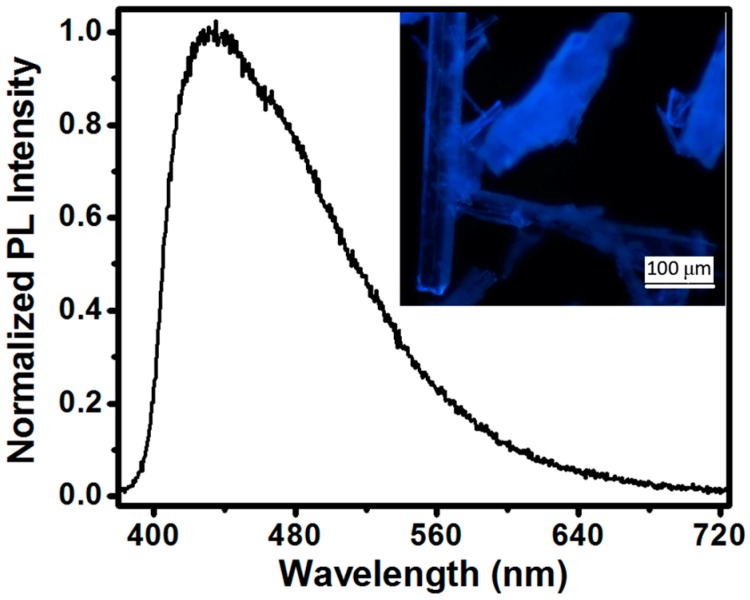
Normalized fluorescence emission spectrum of compound PPDPD in solid state (excitation wavelength 325–365 nm, exposure time 400 ms). PL: photoluminescence.

We further investigated the fluorescence of compound PPDPD in the solid state. As shown in Figure 3, PPDPD emits a strong blue light at λ_max_ 430 nm with a fluorescence quantum yield of up to 41.3%. The inset photograph was taken at an excitation wavelength of 325–365 nm with an exposure time of 400 ms, and shows a strong blue light. The intramolecular rotation process of compound PPDPD may be impeded in the solid state due to the presence of a C-H···F interaction. This may block the non-radiative decay channel and thus provide the observed emission behavior.

The ground-state geometry of compound PPDPD was optimized via hybrid density functional theory (B3LYP) with a 6−31G* basis set using the Gaussian 03 program package. As shown in Figure S9a,b, the highest occupied molecular orbital (HOMO) is a π orbital and the electron density is spread across the whole molecule, while the lowest unoccupied molecular orbital (LUMO) is of π* character and is distributed across the whole molecule except for on the *N*,*N*-diethylaniline group. Thus, a charge-transfer (CT) interaction can occur between the electron donor and electron acceptor groups. As shown in Figure S9c, the phenyl rings at the 1-position and 5-position of the pyrazoline ring exhibited conformational flexibility with dihedral angles of 45° and 60°, respectively, suggesting that the whole molecule possesses a non-planar configuration which may help to impede the π-π stacking interaction in the solid state. 

**Figure 4:**
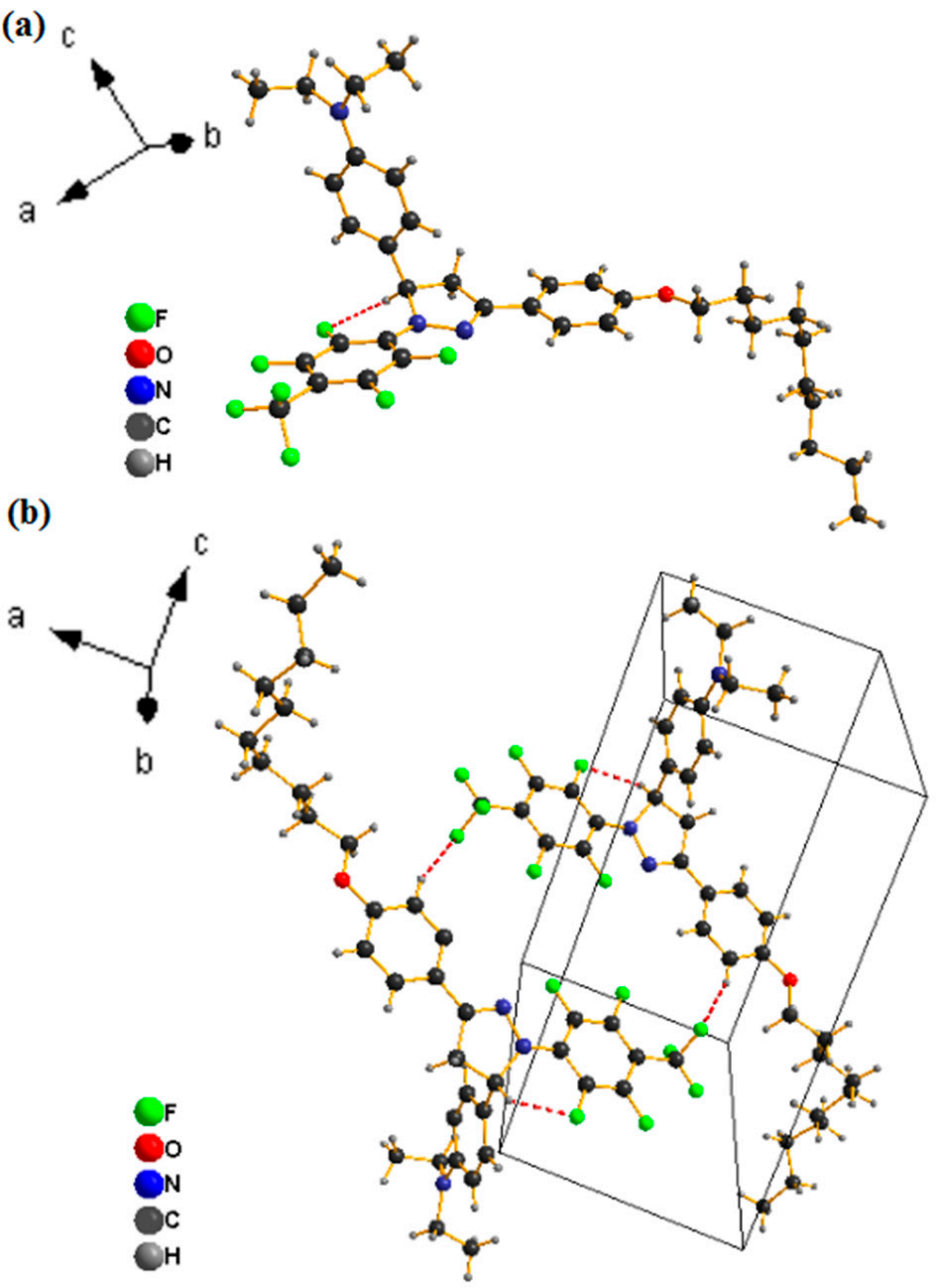
(**a**) Single crystal structure and (**b**) molecular packing diagram of compound PPDPD.

To gain a clear understanding of the correlation between the molecular structure and fluorescence properties of PPDPD, single crystals that were suitable for X-ray analysis (CCDC 1563686 contains the supplementary crystallographic data for this paper. These data can be obtained free of charge via http://www.ccdc.cam.ac.uk/conts/retrieving.html (or from the CCDC, 12 Union Road, Cambridge CB2 1EZ, UK; Fax: +44 1223 336033; E-mail: deposit@ccdc.cam.ac.uk)) were obtained from chloroform/ethanol. As shown in Figure 4a, the phenyl rings in the 1-position and 5-position of the pyrazoline ring possess conformational flexibility with dihedral angles of 149° and 54°, respectively, suggesting that the whole molecule has a non-planar configuration which may help to impede the π-π stacking interaction in the solid state. Furthermore, the presence of intra/intermolecular C-H···F bonds in single crystal (Figure 4b) impedes molecular motion which can lead to blocking of the non-radiative decay channel, hence PPDPD would show high emission efficiency in the solid state.

### 2.3. Electric and Thermal Properties of Compound PPDPD

In order to investigate the electrochemical behavior, a cyclic voltammetry measurement of PPDPD was conducted in dichloromethane (DCM) solution with 0.1 M TBAPF_6_ as the electrolyte. As can be seen in Figure S7, PPDPD displays two reversible oxidative wavelengths. According to the following formula: E_HOMO_ = −[4.8 − EFc + Eoxonset] eV, the HOMO energy level is calculated to be −5.17 eV from the onset of the first oxidation peak. Additionally, the LUMO energy level is estimated to be −1.94 eV according to the following formula: E_LUMO_ = E_HOMO_ + Eg^opt^ (Eg^opt^ is estimated to 3.23 eV according to Figure S6).

The thermal property of PPDPD was evaluated using thermogravimetric analyzer under a nitrogen atmosphere. As displayed in Figure S8, the decomposition temperature of PPDPD with a weight loss of 5% is close to 295 °C.

## 3. Conclusions

In summary, we have successfully synthesized a pyrazoline derivative PPDPD. Although PPDPD exhibits a low fluorescence quantum yield (below 1%) in commonly-used solutions and in a THF/water mixture, it displays a high fluorescence quantum yield (41.3%) in the solid state. Single crystal analysis showed that the intramolecular rotation process of PPDPD may be impeded in the solid state due to the presence of C-H···F interactions; this could block the non-radiative decay channel and hence make compound PPDPD emissive.

## Supplementary Materials

Supplementary materials are available online.
