# Supplementary material for: High Solid Fluorescence of a Pyrazoline Derivative through Hydrogen Bonding
Source: Molecules. 2017 Aug 4;22(8):1304. doi: 10.3390/molecules22081304 (PMC6152280; doi:10.3390/molecules22081304)
Supplement: Supplementary file 1 [file molecules-22-01304-s001.pdf]

Supporting Information

High Solid Fluorescence of a Pyrazoline Derivative through Hydrogen Bonding

Liang Zhang,[1] Jie Liu,[2] Junkuo Gao,[3] Feng Zhang[1] and Liang Ding*,[1]

[1]School of Material Science and Engineering, Yancheng Institute of Technology, Yancheng 224051, Jiangsu, China; zhangliang@ycit.edu.cn (L.Z.); zhangfeng@ycit.cn (F.Z.)
[2]State Key Laboratory of Crystal Materials, Shandong University, Jinan, 250100, P.R. China; liujieicm@sdu.edu.cn (J.L.)
[3]College of Materials and Textiles, Zhejiang Sci-Tech University, Hangzhou, 310018, China; jkgao@zstu.edu.cn (J.G.)
* Correspondence: dl1984911@ycit.edu.cn; Tel.: +86-515-8829-8872

Content

Scheme S1. Synthetic route of compound PPDPD.

Figure S1. ^{1}H NMR spectrum of compound PPDPD.

Figure S2. ^{13}C NMR spectrum of compound PPDPD.

Figure S3. ^{19}F NMR spectrum of compound PPDPD.

Figure S4. HR-MS of compound PPDPD.

Figure S5. Normalized fluorescence emission spectra of compound PPDPD in hexane with different concentrations.

Figure S6. Normalized absorption and fluorescence emission spectra of compound PPDPD in dichloromethane solution.

Figure S7. Cyclic voltammograms of compound PPDPD in DCM solution.

Figure S8. TGA cures of compound PPDPD.

Figure S9. Calculated spatial distributions of the (a) HOMO and (b)LUMO levels of compound PPDPD. (c) Optimized ground-state geometry of compound PPDPD.

Figure S10. Crystallographic data and structure refinement parameters of compound PPDPD

Materials

The chemical reagents 4-diethylaminobenzaldehyde, 4'-hydroxyacetophenone and (2,3,5,6-tetrafluoro-4-(trifluoromethyl)phenyl)hydrazine were purchased from TCI (shanghai)and used as received. All other chemical were used as received without further purification.

Methods

^{1}H, ^{13}C and ^{19}F NMR spectra were recorded on Bruker 300/400-MHz spectrometers. MALDI-TOF mass spectrometric measurements were finished on Shimadzu Biotech AXIMA-TOF^{2TM}. The UV-vis and fluorescence spectra were carried out on Shimadzu UV-2501 and RF-5301 spectrophotometer, respectively. Cyclic voltammetry measurements were conducted on a CHI 604E electrochemical analyser with glassy carbon (diameter: 1.6 mm; area: 0.02 cm^2) as a working electrode, and platinum wires as a counter electrode and a reference electrode, respectively. Fc^+/Fc was used as an internal standard. Potentials were recorded versus Fc^+/Fc in a solution of anhydrous acetonitrile with 0.1 M tetrabutylammonium hexafluorophosphate ($TBAPF_6$) as a supporting electrolyte at a scan rate of 100 mV s^{-1}, by empirical formulas, $E_{LUMO/HOMO}$= -[4.8-E_{Fc} + $E_{re/ox}^{onset}$] eV, where E_{Fc}= 0.062 eV (measured in our setup).

Fluorescence microscope measurement. Fluorescence image was obtained using a Nikon Ti-U Inverted Microscope System equipped with a Nikon C-SHG 1 mercury lamp. The exposure time to acquire a bright photo on a fluorescence microscope is 400 ms, with four attenuators (1/4, 1/8, 1/16, and 1/32).

The fluorescent quantum yields in dilute solutions were determined using quinine bisulfate (φ = 54.6% in 0.1 N H_2SO_4) as a standard using the equation:

$$\phi_s = \frac{F_s}{F_r}\frac{n_s^{\,2}}{n_r^{\,2}}\frac{A_r}{A_s}\phi_r \qquad (1)$$

In this equation, subscripts s and r represent the sample and reference, respectively. F is the integral area of the fluorescence spectra, n is the refractive index of the solution, A is the absorbance, ϕ is the fluorescence quantum yield.

Photoluminescence spectra (PL) and fluorescence quantum yield for solid state were collected on an Edinburgh Instruments FLS 980 system spectrofluorimeter equipped with Xe-900 and integrating sphere.

Single-crystal X-ray diffraction. The suitable single-crystals were selected for single-crystal X-ray data collection with a Bruker SMART APEX-II CCD area detector on a D8 goniometer. The data were collected using graphite-monochromated and 0.5 mm-Mono Cap-collimated Mo-Kα radiation (λ = 0.71073 Å) with the ω scan method at room temperature (Form I and Form II) and 100 K (Form III). The data were processed with the SAINT program of the APEX2 software for reduction and cell refinement. Multi-scan absorption corrections were applied by using the SADABS program for area detector. All structures were solved by the direct method and refined by the full-matrix least-squares method on F^2. All non-H atoms were refined anisotropically. Hydrogen atoms were placed in idealized positions and included as riding with Uiso (H) = 1.2 Ueq (C). Crystallographic data and structural refinements are summarized in Table S1.

Synthesis of 3-(4-(diethylamino)phenyl)-1-(4-hydroxyphenyl)prop-2-en-1-one (PHPO)

The solution of acetic acid (60 mL) containing 4-diethylaminobenzaldehyde (3.54 g, 20 mmol) was added into the solution of sulfuric acid (2 mL) including 4'-hydroxyacetophenone (2.72 g, 20 mmol). The obtained mixture was stirred at room temperature for 24 h, and was poured into the ice-water. The pH of the solution was adjusted to neutral with 0.1 mol L^{-1} NaOH. The crude powder was collected by filtration. The pure powder (4.42 g, 15 mmol, yield: 75%) was recrystallized from the mixture solution of ethanol and water.

^{1}H NMR (400 MHz, DMSO-d_6) δ 10.30 (s, 1H), 8.02 (d, J = 8.6 Hz, 2H), 7.65 (d, J = 8.8 Hz, 2H), 7.59 (d, J = 3.4 Hz, 2H), 6.88 (d, J = 8.6 Hz, 2H), 6.69 (d, J = 8.8 Hz, 2H), 3.45 – 3.37 (m, 4H), 1.12 (t, J = 7.0 Hz, 6H).

Synthesis of 1-(4-(decyloxy)phenyl)-3-(4-(diethylamino)phenyl)prop-2-en-1-one (PPPO)

3-(4-(Diethylamino)phenyl)-1-(4-hydroxyphenyl)prop-2-en-1-one (1.47 g, 5 mmol), 1-bromodecane (1.21 g, 5.5 mmol), and potassium carbonate (828 mg, 6 mmol) were dissolved in acetone (50 mL) under N_2 atmosphere. The reaction mixture was stirred and refluxed for 24 h. The resulting mixture was poured into a water-ice bath. The powder (1.57 g, 3.6 mmol, 72 %) was collected by filtration and washed with cooled ethanol.

^{1}H NMR (400 MHz, $CDCl_3$) δ 8.01 (d, J = 8.8 Hz, 2H), 7.77 (d, J = 15.4 Hz, 1H), 7.53 (d, J = 8.4 Hz, 2H), 7.33 (d, J = 15.5 Hz, 1H), 6.95 (d, J = 8.8 Hz, 2H), 6.66 (d, J = 8.4 Hz, 2H), 4.03 (t, J = 6.5 Hz, 2H), 3.41 (dd, J = 13.8, 6.8 Hz, 4H), 1.80 (m, 2H), 1.46 (m, 2H), 1.28 (m, 12H), 1.20 (t, J = 7.0 Hz, 6H), 0.88 (t, J = 6.3 Hz, 3H).

Synthesis of 4-(3-(4-(decyloxy)phenyl)-1-(2,3,5,6-tetrafluoro-4-(trifluoromethyl)phenyl)-4,5-dihydro-1*H*-pyrazol-5-yl)-*N*,*N*-diethylaniline (PPDPD)

1-(4-(decyloxy)phenyl)-3-(4-(diethylamino)phenyl)prop-2-en-1-one (435 mg, 1 mmol), (2,3,5,6-tetrafluoro-4-(trifluoromethyl)phenyl)hydrazine (260 mg, 1.05 mmol) and hydrochloric acid (1.00 mL) were dissolved in ethanol (50 mL). The reaction mixture was stirred and refluxed for 24 h under N_2 atmosphere. Then, the powder (505 mg, 0.76 mmol, 76 %) was collected by filtration and washed with cooled ethanol.

^{1}H NMR (300 MHz, $CDCl_3$) δ 7.68 (d, J = 8.8 Hz, 2H), 7.07 (d, J = 8.6 Hz, 2H), 6.95 (d, J = 8.8 Hz, 2H), 6.55 (d, J = 8.7 Hz, 2H), 5.58 (dd, J = 11.6, 5.2 Hz, 1H), 4.02 (t, J = 6.5 Hz, 2H), 3.73

(dd, J = 17.1, 11.7 Hz, 1H), 3.32 (d, J = 7.0 Hz, 5H), 1.89 – 1.76 (m, 2H), 1.48 (dd, J = 14.6, 7.1 Hz, 2H), 1.30 (s, 12H), 1.14 (t, J = 7.0 Hz, 6H), 0.91 (s, 3H).

^{13}C NMR (75 MHz, $CDCl_3$) δ 160.4, 150.9, 147.8, 127.7, 127.2, 127.0, 124.4, 114.7, 111.7

^{19}F NMR (282 MHz, $CDCl_3$) δ -55.35 (t, J = 21.1 Hz, 3H), -142.84 – -143.12 (m, 2H), -148.56 (d, J = 12.7 Hz, 2H).

HR-MS, Formula, $C_{36}H_{43}N_3OF_7$, Calc. Mass 666.3294, Found 666.3309.

Cyclic voltammetry measurement of compound PPDPD has been performed in DCM solution (with 0.1 M $TBAPF_6$ as the electrolyte) to investigate its electrochemical behavior. As shown in Figure S7, compound PPDPD in DCM solution exhibits two reversible redox process. The highest occupied molecular orbital (HOMO) energy level of compound PPDPD was estimated to be -5.17 eV from the onset of the first oxidation potential with reference to Fc^+/Fc (-4.8 eV) using the equation of $E_{HOMO} = -[4.8 - E_{Fc} + E_{ox}^{onset}]$ eV. The lowest unoccupied molecular orbital (LUMO) energy level of compound PPDPD was estimated to be -1.94 eV according to the equation of $E_{LUMO} = E_{HOMO} + E_g^{opt}$ (E_g^{opt} = 3.23 eV from Figure S6).

The thermal property of compound PPDPD was evaluated by TGA under nitrogen atmosphere. As shown in Figure S8, compound PPDPD exhibits very good thermal stability with an onset decomposition temperature of ~ 295 °C (considering the 5% weight loss temperature).

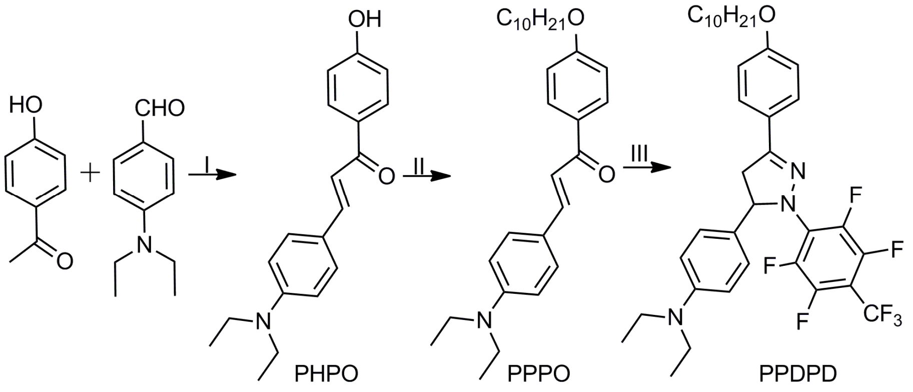

Scheme S1. Synthetic route of compound PPDPD. (I) H_2SO_4/CH_3COOH, 75%; (II) 1-bromodecane/potassium carbonate/acetone, 72%; (III) (2,3,5,6-tetrafluoro-4-(trifluoromethyl)phenyl)hydrazine/HCl/ethanol, 76%.

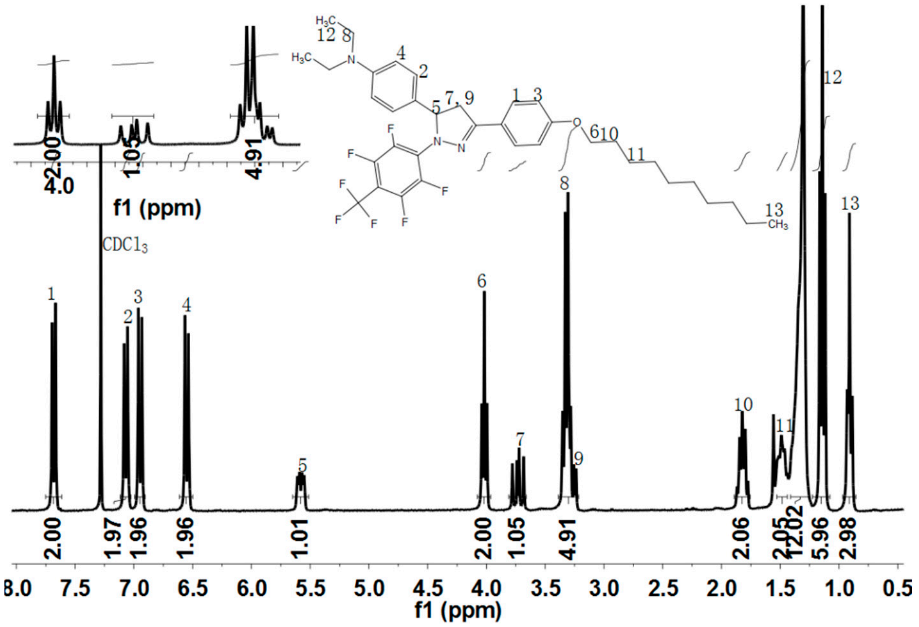

Figure S1. ^{1}H NMR spectrum of compound PPDPD.

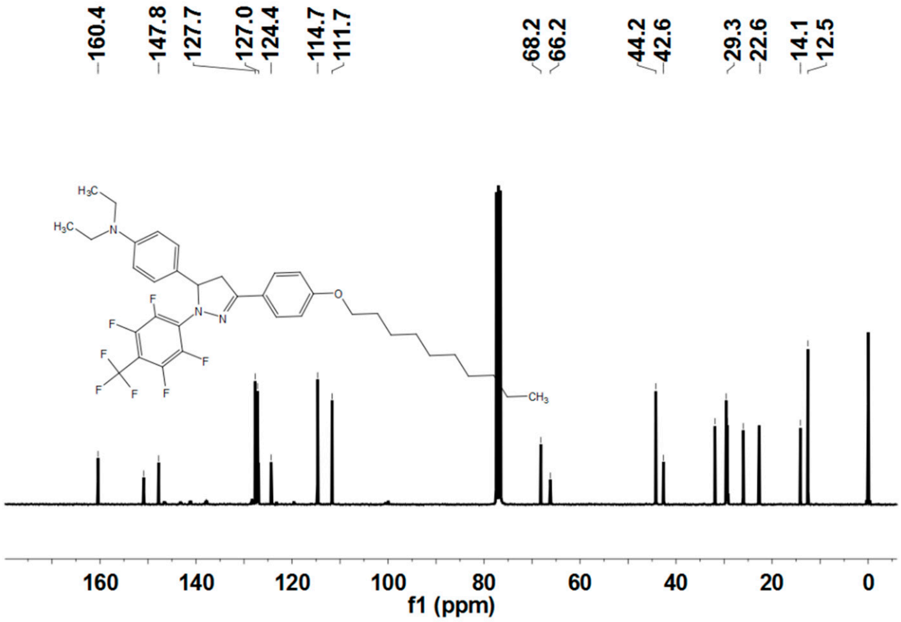

Figure S2. ^{13}C NMR spectrum of compound PPDPD.

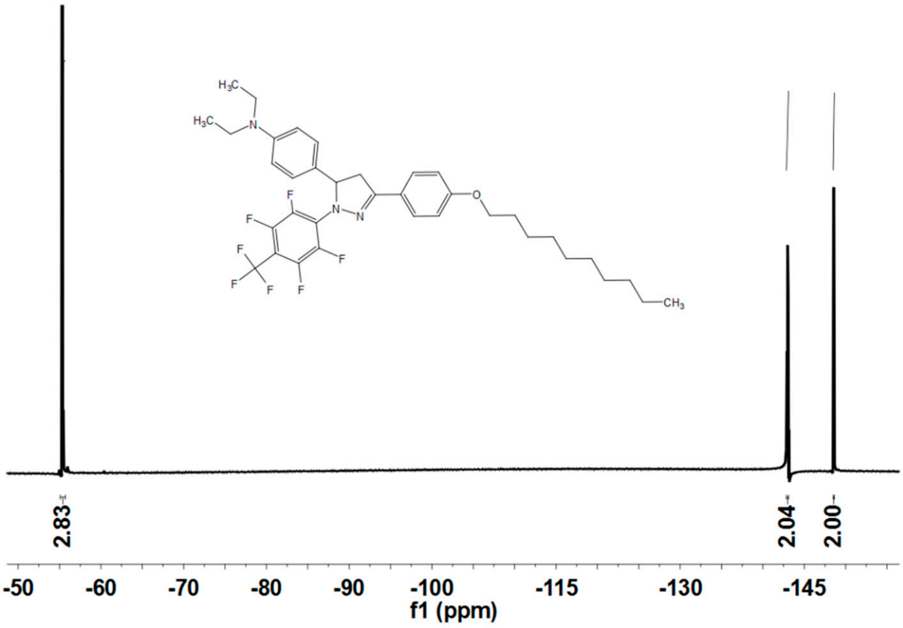

Figure S3. ^{19}F NMR spectrum of compound PPDPD.

Elemental Composition Report

Single Mass Analysis

Tolerance = 5.0 PPM / DBE: min = -3.0, max = 70.0
Element prediction: Off
Number of isotope peaks used for i-FIT = 3

Monoisotopic Mass, Even Electron Ions
83 formula(e) evaluated with 1 results within limits (all results (up to 1000) for each mass)
Elements Used:
C: 18-36 H: 15-43 N: 0-3 O: 0-3 F: 1-7
C36H42F7N3O
G7f 10 (0.239)

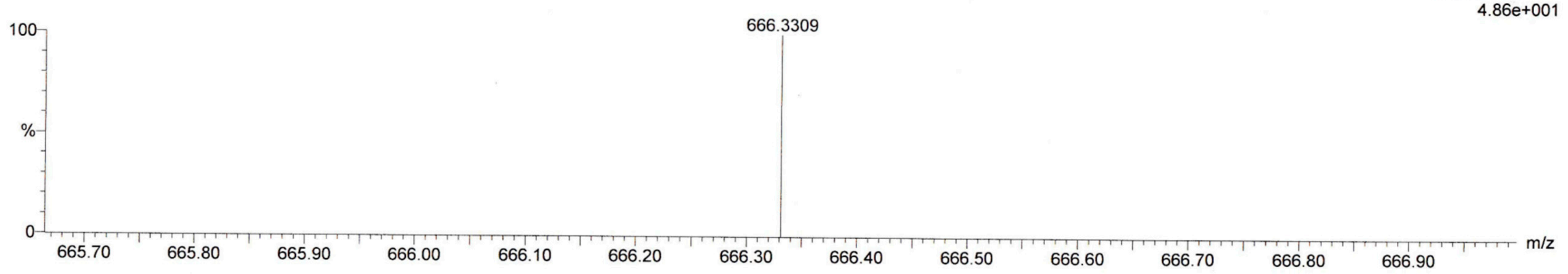

Minimum:				-3.0			
Maximum:		5.0	5.0	70.0			
Mass	Calc. Mass	mDa	PPM	DBE	i-FIT	i-FIT (Norm)	Formula
666.3309	666.3294	1.5	2.3	13.5	18.1	0.0	C36 H43 N3 O F7

Figure S4. HR-MS of compound PPDPD.

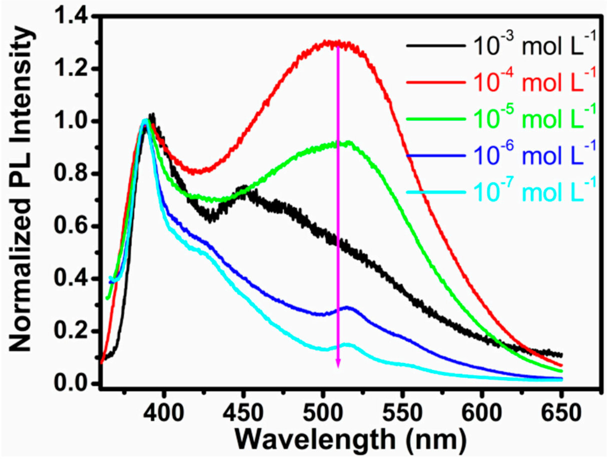

Figure S5. Normalized fluorescence emission spectra of compound PPDPD in hexane with different concentrations.

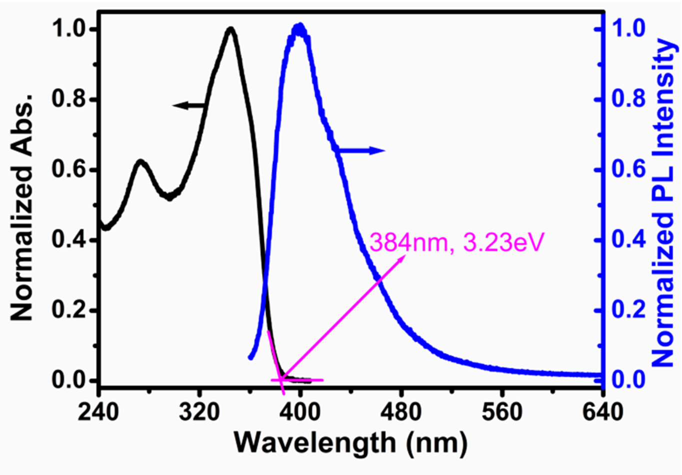

Figure S6. Normalized absorption and fluorescence emission spectra of compound PPDPD in dichloromethane solution.

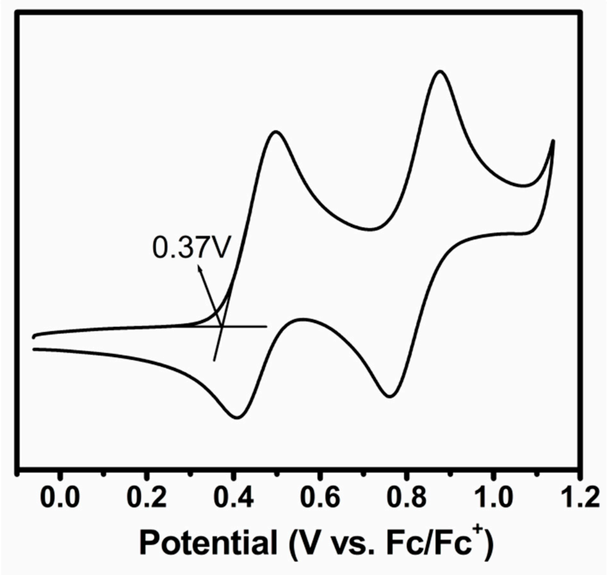

Figure S7. Cyclic voltammograms of compound PPDPD in DCM solution.

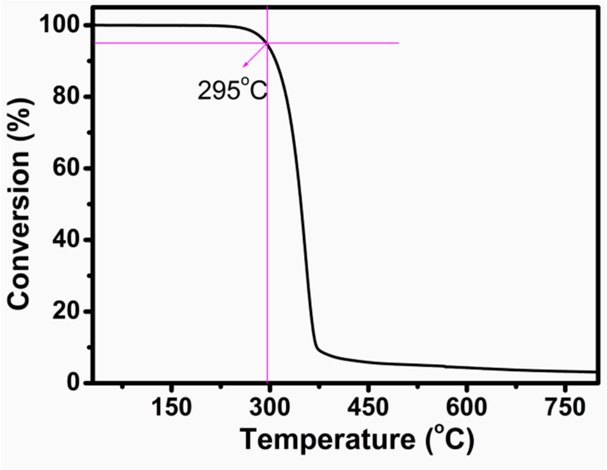

Figure S8. TGA cures of compound PPDPD.

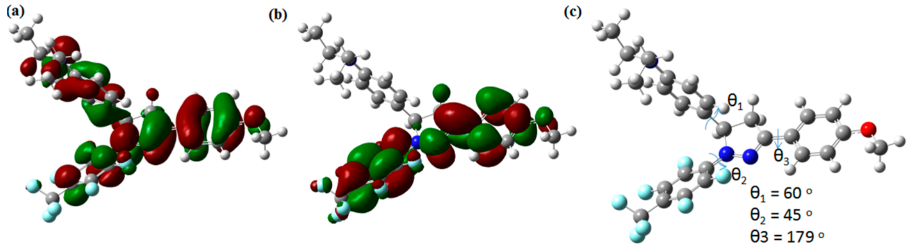

Figure S9. Calculated spatial distributions of the (a) HOMO and (b)LUMO levels of compound PPDPD. (c) Optimized ground-state geometry of compound PPDPD.

Bond precision: C-C = 0.0065 A Wavelength=0.71073

Cell:	a=8.8449(8)	b=12.8119(11)	c=16.2966(14)
	alpha=104.413(1)	beta=90.251(1)	gamma=103.126(1)
Temperature:	296 K		

	Calculated	Reported
Volume	1738.2(3)	1738.2(3)
Space group	P -1	P-1
Hall group	-P 1	?
Moiety formula	C36 H42 F7 N3 O	?
Sum formula	C36 H42 F7 N3 O	C36 H42 F7 N3 O
Mr	665.73	665.73
Dx,g cm-3	1.272	1.272
Z	2	2
Mu (mm-1)	0.103	0.103
F000	700.0	700.0
F000’	700.43	
h,k,lmax	11,16,20	11,16,20
Nref	7661	7615
Tmin,Tmax	0.959,0.971	0.951,0.971
Tmin’	0.951	

Correction method= # Reported T Limits: Tmin=0.951 Tmax=0.971
AbsCorr = ?

Data completeness= 0.994 Theta(max)= 27.100

R(reflections)= 0.0920(4570) wR2(reflections)= 0.3429(7615)

S = 1.107 Npar= 427

Figure S10. Crystallographic data and structure refinement parameters of compound PPDPD.
